# Attitude towards seeking professional help for mental health among medical students In Karachi, Pakistan

**DOI:** 10.2144/fsoa-2023-0114

**Published:** 2024-05-16

**Authors:** Mubashir Zafar, Tafazzul H Zaidi, Nadira H Zaidi, Muhammad WN Ahmed, Sobia Memon, Faheem Ahmed, Amal Siddiqui, Abeen Fatima, Ayesha Shahid, Balaj Hussain, Anosha Salam

**Affiliations:** 1 Family and Community Medicine Department, College of Medicine, University of Hail, Hail, Kingdom of Saudi Arabia; 2 Department of Community Medicine, Sindh Medical College, Jinnah Sind Medical university, Karachi, Pakistan; 3 Shaheed Zulfiqar Ali Bhutto Institute of Science & Technology, Karachi, Pakistan

**Keywords:** counseling, medical students, mental health, professional help, stigma

## Abstract

**Aim:** In Pakistan, seeking help for mental health is considered a social stigma and a large number of medical students are suffering from mental health. This study aimed to investigate the attitude toward seeking professional care for mental health issues among medical students. **Methodology:** A cross-sectional study was conducted with a total of 316 students selected through multistage stratified cluster sampling. With each academic year 500 students were enrolled. Linear regression analysis was used to find the association of outcome and independent variables. **Results:** Around 56% of students had a negative attitude toward seeking professional help. Common predictors associated with a negative attitude were age (p < 0.001), academic year (p < 0.001) and with self-harm behavior (p < 0.001). **Conclusion:** University students generally had moderate intentions to seek counseling regarding mental health.

## Plain language summary

Mental health disorders are considered common in society. In particular, medical students commonly suffer from depression and anxiety. This study found that there are different factors that contribute to a negative attitude towards seeking mental health counseling. For example, a younger age and a history of self-harm contributed the most to a negative attitude towards mental health help, and not sharing any health problems with parents and doctors. There is a need to increase awareness among students regarding mental disorders and seeking help to overcome problems.

## Abstract (plain language summary)

A total of 18% of students were diagnosed with a mental disorder.

Common factors were the presence of a confiding relationship (78.8%), sharing secret with someone (93%), thoughts of self-harm (28.2%), social stigma (59.8%) as the major barrier to seeking mental health help.

44% of participants had a negative attitude toward professional help regarding mental health.

The WHO characterises mental health as an important aspect of overall well-being, noting that it is more than the absence of mental disorders. Therefore, the promotion, protection and restoration of mental health should be considered a critical concern at all levels [1]. There are various types of mental disorders, characterized by a mixture of abnormal thoughts, perceptions, emotions, behavior and interpersonal connections. Among all the mental disorders, depression is the most common and leading cause of disability worldwide and globally, affecting 264 million people [2].

According to the research, people with mental disorders have a greater mortality rate and approximately 8 million deaths per year worldwide [3]. Mental health is not a priority in the developing world when compared with physical health. Social stigma associated with mental health difficulties causes embarrassment and shame, as the public may perceive sufferers as being incapable, unsociable, irresponsible, fearful, or pitiful [4]; and as a result those affected are less likely to seek treatment for this mental disorder [5]. There are a number of different outcomes stemming from receptivity to social stigma, with suicidal ideation being the most important. Commonly used terms associated with this stigma are ‘dangerous’, ‘unpredictable’, ‘incompetent’, ‘dependent’ and ‘not curable’. These words are frequently used by medical students to describe mental illness. A previous study found an association between mental health stigma and a lower likelihood of seeking professional care [5]. In developed regions such as Europe or the USA, a significant treatment gap exists, with approximately two thirds of those in need of care for mental health not receiving proper care, which shows that not only is it stigmatizing to be mentally ill, but it is also stigmatizing to seek (professional) aid, which hinders people from seeking therapy [6].

According to the National Institute of Mental Health (NIMH) in the USA, depression is most common among students [7], and can lead to suicidal ideation [8].

Professional counseling is a major component for secondary prevention of mental disorder, but it is evident that approximately half of adults in the USA do not seek professional help in order to overcome these problems [9,10].

Prior studies have shown that young people display a high risk of mental disorders, yet a help-seeking attitude is rare amongst younger people, people with lower levels of education, and people from low socioeconomic backgrounds [10,11]. Nowadays, mental disorders are most common in college and university students. There are several risk factors for the development of mental issues in students, including academic considerations, social factors, psychological risk factors, lifestyle factors and physiological aspects [12].

Mental health literacy (MHL) had a direct impact on attitudes, which were favorably predicted by social support. To encourage a favorable situation in which a person seek professional help for mental issue, the findings imply that MHL and family support overcome the mental disorders. Mental health education for those at risk of mental illness and their families, who are likely to impact the attitude toward seeking assistance, could be beneficial [13].

To the best of our knowledge, there has been no previous study conducted in order to determine the kinds of professional help regarding mental health among medical students. In Pakistan, mental disorder is associated with social stigma. Mental disorder is also common among medical students in Pakistan due to the burden of studies, and social and economic problems. Our research results may help to remove the barriers to seeking professional help regarding mental health among medical students, and objective of this study is to discover the attitude levels toward seeking professional help for mental health issues in Karachi, Pakistan.

## Materials & methods

### Study setting

Medical college located in the city of Karachi, total enrolment of students around 2500. Each year, 500 students are enrolled. Bachelors of Medicine and Bachelors of Surgery programme ran for a total of 5 academic years. The study was conducted from December 2022 to April 2023.

### Inclusion & exclusion criteria

Students aged between 18 and 27 years were included in the study, any participants who refused to give informed consent were excluded from the study.

### Study variables

Outcome variables were level of attitude. Independent variables were age, gender, academic year of study, barrier to seek help for mental health, confiding relationship, self-harm.

Each student was interviewed by the investigators, who were adequately trained to minimize investigator bias.

### Study design & subjects

The design of this study was cross-sectional, and participants of this study were medical students, selected through multistage stratified cluster sampling. The World Health Organization (WHO) sample size calculator for health studies software was used to calculate the sample size with a parameter of a 5% margin of error with 95% CI and assuming an 11% prevalence of attitude toward professional help [14], 5% bond-on error and 10% non-response rate. The required sample size is 316.

### Study participants selection

After getting permission from the Dean of Colleges, questionnaires were distributed amongst study participants, including a written consent form, through social media platforms. it was requested that study participants returned the forms within a week. Data was screened on a weekly basis for omission of error and stored in a safe place.

### Study tools

A validated and structured questionnaire was used. It comprised of three sections: the first section pertained to the socio-demographic characteristic of study participants, the second section pertained to the measurement of current mental health symptoms and validated self-report scale of attitudes toward seeking professional psychological help scale-short form (ATSPPH-SF) [15]. This scale reliability and validity were determined through different studies [15,16], and Cronbach's s alpha coefficients and test reliability were 0.83 and 0.86, respectively. The scale comprised of ten items, with each item rated on a Likert scale comprising of four points: 0 (strongly disagree), 1 (disagree), 2 (agree) and 3 (strongly agree). Scores were added and higher scores indicated a positive attitude toward professional help. Items 2, 4, 8, 9 and 10 reversed the scores, e.g.: 0 (strongly agree) and 3 (strongly disagree). Items 1, 3, 5, 6, 7 were added as per the original points. This study found a Cronbach's alpha value of 0.85.

### Analysis of data

Epi-data software and Statistical Package of Social Science Software program (SPSS) version 26.0 software (IBM SPSS Statistics for Windows, Version 26.0. Armonk, NY: IBM Corp) were used for data entry and analysis of data respectively. Mean and standard deviation were calculated in descriptive statistics. Inferential statistical analysis used determine the association of attitude toward professional help and social-demographic characteristics. Statistical significance was determined by p-value by <0.05.

## Results

The mean age of study participants was 21 years of age (SD ± 0.50). Most (83.5%) participants were female students and 47.8% of students from the fourth academic year (Table 1).

**Table 1.** Socio-demographic characteristics and mental health of study participants (n = 316).

Characteristics	Frequency (n) (%)
Age (years, mean ± SD)	21 ± 0.500
^18-21^	162 (51.3)
^22-25^	154 (48.7)
Gender	
– Female	264 (83.5)
– Male	52 (16.5)
Academic year of study	
– First	50 (15.8)
– Second	40 (12.7)
– Third	48 (15.2)
– Forth	151 (47.8)
– Fifth	27 (8.5)
Mental health among study participants	
Confiding relationship with anyone	
– Yes	249 (78.8)
– No	67 (21.2)
Sharing secrets or private matter to others	
– Some of time	294 (93)
– Every time	22 (7)
Any time to think self-harm	
– Yes	89 (28.2)
– No	227 (71.8)
Barriers to seek mental health help	
– Social stigma	189 (59.8)
– Affordability of treatment	21 (6.6)
– Family members	106 (33.5)
Practice to seek professional advise if mental disorder diagnosed	
– Help form mental health professionals	150 (47.5)
– Help from faith healers	22 (7)
– Help from family members/friends	144 (45.5)
Rate your mental health	
– Poor	57 (18)
– Average	103 (32.6)
– Good	156 (49.4)
Mental disorder diagnosed (medical verified)	
– Yes	57 (18)
– No	259 (82)

The mental health of study participants was: presence of a confiding relationship 78.8%, sharing a secret with someone 93%, thoughts of self-harm 28.2%, social stigma (59.8%) is a major barrier to seeking help for mental health issues, and 18% of participants were diagnosed with a mental disorder during the study (Table 1).

In regression analysis, results found that 24% of variability in attitude toward professional help was determined by mental health condition. After adjustment of covariates in regression analysis, both attitude levels negatively related to mental health conditions. Every 1% increased the age and barrier to seek mental health help among study participants, 1% and 4% decreased the attitude level, respectively (Table 2).

**Table 2.** Prediction of intention to seek professional psychological help among study participants.

Characteristics	β (95% CI) (p-value)	R-square change (R^2^)
Age	0.070 (-0.61–1.45) (0.001)	0.24
Gender	-0.015 (-1.03–0.79) (p < 0.081)	0.24
Academic year of study	0.133 (0.762–0.115) (0.001)	0.24
Self-harm	-0.047 (-1.14–0.511) (0.001)	0.24
Barrier to seek medical help	-0.088 (-0.66–0.09) (0.001)	0.24
Advise to mental disorder person	0.020 (-0.46–0.66) (0.001)	0.24

**Figure 1.**
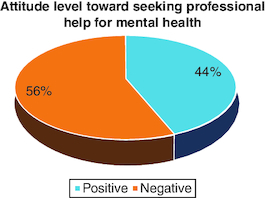
Attitude level toward seeking professional help for mental health.

44% of participants held negative attitudes toward professional help regarding mental health (Figure 1).

## Discussion

The results of this study showed that medical students hold a moderate score on stigmatization levels.

In this study, around 83.5% of participants were female and 16.5% were male, which was similar to the findings of the study, conducted by Chew-Graham CA *et al.*, among college students in which 67.5% were females and 32.5% were males [17].

In this study it was found that amongst participants who have a confiding relationship 43% marked parents, 19.3% marked friends, 13.6% marked siblings, 21.2% chose no one and others went with the option ‘someone else’. These study results were similar to other study results in which students prefer to seek help from family and friends. This study showed that 57% of medical students used self-isolation as their stress coping mechanism, 56.3% get enough sleep, 30.1% participate in social activities, 24.4% do exercises, 23.1% reconnect with their old friends and 11.4% make new friends [18,19]. This was contrary to the findings in the University of Alaska Anchorage study, whose results found that family and friends help for coping with mental disorder get significant improvement and some students overcome this problem through leisure activities such as visiting restaurants and going to cinema [20].

This study results found that among the barriers against seeking help for mental health problems, 59.8% reported stigma as the major barrier, 50% reported mental health literacy, 44.6% reported family members, 39.6% reported expensive treatment and 16.5% reported autonomy. This was contrary to the findings among college students, of which 66% reported concerns about ‘perceptions’ that mental disorder was a minor issue and would be fixed after several months of study in the college; alternatively, they cited a lack of time to visit counseling due to busy schedules [18]. Some students reported that their major obstancle to seeking help was the belief that they can fix issues through the use of self help, and that they do not need treatment due to their problems being minor or transient in nature. A lack of time was the second most frequently mentioned barrier, with 26.8% of students feeling that they are too busy or have more pressing priorities that get in the way of reaching out for professional help. Furthermore, nearly 18% of the sample expressed their preference for managing. Another major barrier for seeking help is the social stigma in the society [21,22].

This study showed that only 18% of the medical students have been diagnosed and 82% reported no diagnosis. This result was similar to the findings of a study of medical students in the USA where only 28.1% indicated having diagnosed with a mental disorder and the remaining 79.9% reported no diagnosis [23].

Furthermore, 24% of attitude variability was due to a mental health condition. Age and barrier to seek professional help regarding mental health were negatively associated with attitude level. This was contrary to the findings in a study at Northwest Victoria, where 88.1% agreed that age and barrier were positively associated with attitude. Regarding priority for seeking help, only, 41.1% agreed, 9.8% strongly agreed, 36.7% disagreed and 12.3% strongly disagreed that seeking help was a priority [24,25].

### Limitations of the study

This study has several limitations. Firstly, self-reported data may be subject to recall and social desirability biases, leading to inaccurate information. Secondly small sample sizes and may not be representative of the entire population. Moreover, cross-sectional studies provide only a snapshot of knowledge, attitudes and practices at a specific time, limiting their ability to capture long-term changes. Despite these limitations, cross-sectional studies remain valuable in providing insights into PID-related factors, but should be complemented with other research methods for a comprehensive understanding.

## Conclusion

Study found that statistically significant negative relationship with attitude level and stigma for seek professional help toward mental health. Though an adequate proportion of undergraduate medical students believe that one should get professional help for mental health, a lot needs to be done to change people's perception about stigmatization of mental health and to promote mental health.
